# Factors influencing and changes in childhood vaccination coverage over time in Bangladesh: a multilevel mixed-effects analysis

**DOI:** 10.1186/s12889-023-15711-x

**Published:** 2023-05-11

**Authors:** Satyajit Kundu, Subarna Kundu, Abdul-Aziz Seidu, Joshua Okyere, Susmita Ghosh, Ahmed Hossain, Najim Z. Alshahrani, Md. Hasan Al Banna, Md. Ashfikur Rahman, Bright Opoku Ahinkorah

**Affiliations:** 1 Global Health Institute, North South University, Dhaka, 1229 Bangladesh; 2 Faculty of Nutrition and Food Science, Patuakhali Science and Technology University, Patuakhali, 8602 Bangladesh; 3 Statistics Discipline, Khulna University, Khulna, 9208 Bangladesh; 4 Faculty of Built and Natural Environment, Department of Estate Management, Takoradi Technical University, Takoradi, Ghana; 5 Centre for Gender and Advocacy, Takoradi Technical University, Takoradi, Ghana; 6 College of Public Health, Medical and Veterinary Sciences, James Cook University, Townsville, QLD Australia; 7 Department of Population and Health, University of Cape Coast, Cape Coast, Ghana; 8 Department of Food Technology and Nutrition Science, Noakhali Science and Technology University, Noakhali, Bangladesh; 9 Department of Nutrition Science, Purdue University, West Lafayette, IN 47907 USA; 10 College of Health Sciences, University of Sharjah, Sharjah, 27272 United Arab Emirates; 11 Department of Public Health, North South University, Dhaka, 1229 Bangladesh; 12 Department of Family and Community Medicine, Faculty of Medicine, University of Jeddah, Jeddah, 21589 Saudi Arabia; 13 Nutrition Initiative (NI), Kushtia, Bangladesh; 14 Development Studies Discipline, Khulna University, Khulna, 9208 Bangladesh; 15 School of Public Health, Faculty of Health, University of Technology Sydney, Sydney, Australia

**Keywords:** Childhood vaccination, Immunization, Prevalence, Demographic and Health Survey, Bangladesh

## Abstract

**Introduction:**

This study aimed to investigate the associated factors and changes in childhood vaccination coverage over time in Bangladesh.

**Methods:**

Bangladesh’s Demographic and Health Surveys from 2011, 2014, and 2017-18 provided data for this study on vaccination coverage among children aged 12 to 35 months. For three survey periods, multilevel binary logistic regression models were employed.

**Results:**

The overall prevalence (weighted) of full vaccination among children aged 12–35 months were 86.17% in 2011, 85.13% in 2014, and 89.23% in 2017-18. Children from families with high wealth index, mothers with higher education, and over the age of 24 and who sought at least four ANC visits, as well as children from urban areas were more likely to receive full vaccination. Rangpur division had the highest change rate of vaccination coverage from 2011 to 2014 (2.26%), whereas Sylhet division had the highest change rate from 2014 to 2017-18 (34.34%).

**Conclusion:**

To improve immunization coverage for Bangladeshi children, policymakers must integrate vaccine programs, paying special attention to mothers without at least a high school education and families with low wealth index. Increased antenatal care visits may also aid in increasing the immunization coverage of their children.

## Introduction

Vaccinations are widely acknowledged as one of the safest and most cost-effective ways to protect children against infectious diseases such as tuberculosis and measles [1]. Thus, childhood vaccination has been increasing over the past decades [2]. Evidence shows that vaccination of children against diphtheria-tetanus-pertussis (DTP3) increased astoundingly from a global coverage of 20% in 1980 to 85% in 2019 [3]. More profound is the evidence that vaccination averts between 2 and 3 million deaths attributable to vaccine-preventive diseases such as Diphtheria, Pertussis, Tetanus, and Measles among children under-five every year [4, 5].

Although the world has seen remarkable improvements in childhood vaccination, achieving complete coverage over time remains an important public health concern [6]. Not every child is getting vaccinated. For instance, 19.4 million infants did not receive basic vaccination as at the end of 2019 [7, 8]. Most of these deficiencies in childhood vaccination coverage are recorded in low-and-middle-income countries (LMICs). The WHO asserts that, in remote rural areas of LMICs, only 1 out of 20 children have access to vaccination [9]. The consequences of not achieving complete childhood vaccination cannot be underestimated. Vaccination provides an opportunity to avert millions of deaths and a host of vaccine-preventable diseases among children [1]. Within the framework of the WHO, children who miss scheduled vaccinations for any reason due to health facility problems such as canceled vaccination schedules or vaccine stock-outs are categorized as having incomplete vaccination [10]. Denying children access to a complete dose of vaccines would be catastrophic as a countless number of children will die or develop some form of disabilities [11]. As such, it is imperative to understand the nuances that characterize childhood vaccination coverage over time.

Available evidence suggests that there are several factors that influence the uptake of vaccination for children under-five. For instance, a qualitative study by Jalloh et al. [12] indicates that perceived beliefs about the side effects coupled with concerns about receiving multiple vaccines on the same day were significant barriers to the uptake of childhood vaccination and its coverage. Also, other studies from South East Asia [13] have shown that maternal age, wealth status, and frequency of antenatal care visits are associated with the likelihoods of complete childhood vaccination coverage.

Since 1979, the Government of Bangladesh has started vaccinations against six preventable diseases (tuberculosis; diphtheria, pertussis, and tetanus; polio; and measles) through the Expanded Program on Immunization (EPI) [14]. According to the Bangladesh Immunization guidelines, children who have received one dose of the vaccine against tuberculosis, Bacille Calmette-Guerin (BCG), three doses of a pentavalent vaccine (DPT, Hib, and HepB), three doses of the polio vaccine (excluding the polio vaccine given at birth), and one dose of the measles and rubella vaccine are considered as fully vaccinated, if they would miss any of the recommended doses they will be considered as partially vaccinated [14, 15].

Bangladesh as a country has attained significant heights in reducing childhood mortality; this is seen in the country’s capacity to meet the Millennium Development Goal 4 [16]. Through the implementation of the WHO’s Expanded Programme on Immunization (EPI), Bangladesh was able to commit sufficiently towards the promotion of childhood vaccination coverage which saw a sustained impact on childhood (under the age of 5 years) mortality, reducing it from 133 deaths per 1000 live births in 1993 to 27 deaths per 1000 live births in 2021 [16, 17] which is projected to further reduce to 17.6 deaths per 1000 live births by 2030 [18]. In a bid to augment efforts toward childhood vaccination coverage, the Bangladeshi government came up with different immunization programs; for instance, nationwide supplementary immunization activities (immunization campaigns and case-based surveillance system ) were executed from 2000 to 2016 in order to eliminate measles from the country [19]. These initiatives resulted in a significant decline in the incidence of measles, from 14,745 incident cases in 2010 to 972 in 2016 [12, 19]. Nevertheless, in 2015, Bangladesh was reported to have a higher number of under-five mortality with 119 deaths accounted for 2% share of global under-five deaths in 2015 which placed the country among the top ten countries with the highest number of under-five mortality, with vaccine-preventable diseases being the causes of these mortalities [12, 20]. This makes Bangladesh an opportune context to understand childhood vaccine coverage and its concomitant factors.

Bangladesh relies on composite estimates based on administrative coverage data gathered from healthcare providers, population-based household surveys, and governmental agencies [16]. However, due to the incompleteness and mistakes associated with the original collection of data on childhood immunization in Bangladesh, such estimates are frequently incorrect [16]. As a result, utilizing a nationally representative survey provides much more clarity and strong data to investigate the factors that influence vaccination coverage [21]. The only study in Bangladesh that used a nationally representative data and also investigated the trends and determinants of vaccination coverage limited their analysis to 2014 [21]. However, we postulate that between 2014 and the latest demographic and health survey (i.e., 2017-18), there would have been some significant changes and policy reforms that may cause changes in the determinants of vaccination coverage in the country. Moreover, none of the existing studies performed a geospatial analysis to understand the geographical spread and disparities in childhood vaccination coverage in Bangladesh. This presents a knowledge gap in terms of understanding of the current determinants of vaccination coverage in Bangladesh. Hence, we were motivated to fill this knowledge gap in the understanding of the changes in the vaccination coverage and its associated factors overtime. Using nationally representative data from 2011, 2014, and 2017-18 Bangladesh Demographic and Health Survey, the study aims to track the vaccination status of children aged 12 to 35 months and examine the factors that influence full immunization coverage in Bangladesh.

## Methods

### Data source and study design

**Fig. 1:**
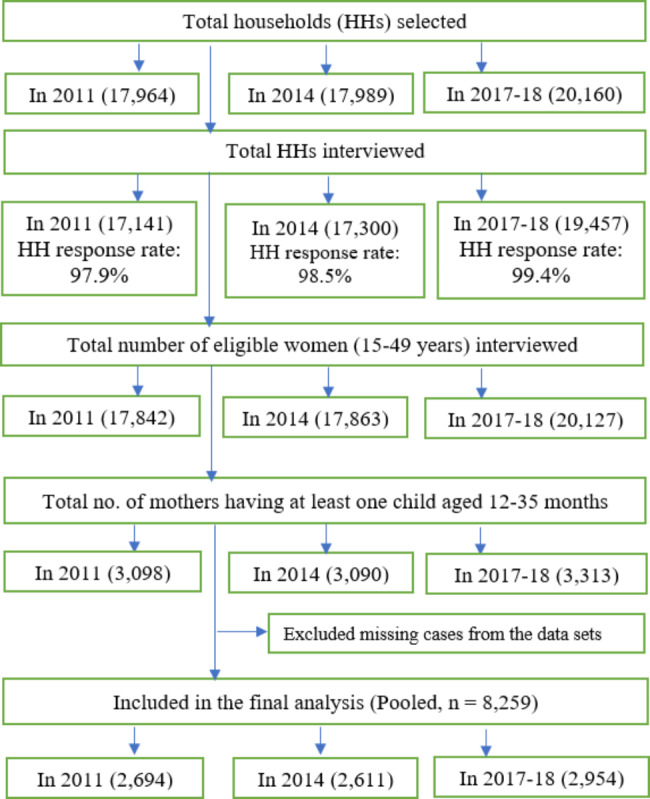
Flow chart of the participants selection from the Bangladesh Demographic and Health Survey (BDHS 2011, 2014, and 2017 − 18) data

The current study utilized three recent nationally representative cross-sectional Bangladesh demographic health survey data (2011, 2014, and 2017-18 BDHS). The survey included both urban and rural households from all administrative regions of Bangladesh. The data were collected using two-stage stratified cluster sampling design of the household. At the first stage, enumeration areas (EAs) were selected with probability proportional to sizes like 672 in 2017-18, and 600 in both 2014 and 2011 BDHS respectively. After getting the EAs (cluster), on average 30 households were selected from each cluster using systematic sample selection. Detailed information on the sampling design could be found in the BDHS survey reports [15, 22, 23]. The final sample included in the analysis was 2,694 participants from BDHS 2011, 2,611 participants from BDHS 2014, and 2,954 participants from BDHS 2017-18. The detailed procedure of participants’ selection from three periods of BDHS has been shown in Fig. 1.

### Outcome measure

**Fig. 2:**
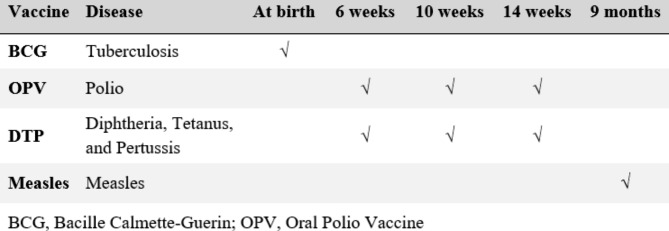
Basic vaccination administration schedule for children under 12 months in Bangladesh

Vaccination status among children aged 12–35 months was assessed and previously similar studies were also conducted among children of the same age range (12–35 months)[24–27]. The following four basic vaccines for children were considered in this study: Bacille Calmette-Guérin (BCG vaccine); diphtheria, pertussis, and tetanus (DPT vaccine); poliomyelitis (oral polio [OPV] vaccine); and measles (measles vaccine) [22]. Children aged 12–35 months were considered to be fully vaccinated if they got the BCG vaccine at birth, three doses of polio, three doses of DPT and one dose of measles at any time before the survey (Fig. 2). Partially vaccinated were defined as lacking any dose of the basic vaccination. While those who failed to take the recommended doses of vaccine were categorized as “none”. Vaccination coverage information was collected in two ways from the vaccination card or from the mother’s verbal report. For final analysis, vaccination status was dichotomized as “fully vaccinated” and “not fully vaccinated” (merging partially vaccinated and no vaccinated). Hepatitis B vaccine (1–3 dose), *Haemophilus influenzae* type B vaccine, and inactivated polio vaccine (IPV) were not included in the current study since information on these were not available in 2011 and 2014 BDHS.

### Explanatory variables

According to the guidance of reviewed literature and the availability of the variables, several demographic and health variables were included in this current analysis [1, 28, 29]. The included variables for this study are child’s age, mother’s age, mother’s education, antenatal care (ANC) visit, place of residence, division, gender, place of delivery, and number of children. Children aged 12–35 months were selected to conduct the study who were categorized as “12–23 months”, “24–35 months”. Maternal age was categorized as “less than 24 years”, “24–34 years”, and “above 34 years” [30, 31]. The household wealth index was calculated using principal component analysis of the household characteristics and different household assets [23]. The wealth index of the household was recoded as “poor” (poorest or poorer), “middle”, and “rich” (richer, richest) [32]. Media access was measured by asking mothers about the number of times they read a newspaper, listen to the radio, and watch television. Adding these variables media access was recoded as “less than once a week” or “at least once a week”. Place of delivery was recoded as “home”, “health facility delivery” (public and private health care facility), and “others” (don’t know, didn’t response, and missing). Antenatal care visits of mothers were categorized into “no visit”, “1–3 visits”, “4 or more visits”, and “others”. Maternal education was classified into “no education”, “primary”, “secondary” and “higher” education. Currently, Bangladesh has eight administrative divisions including Barisal, Dhaka, Chittagong, Khulna, Mymensingh, Rajshahi, Rangpur and Sylhet. However, Mymensingh division was separated from Dhaka division in 2015 [33]. That’s why information of Mymensingh division was not available separately in 2011 and 2014 BDHS survey, and we merged the Mymensingh and Dhaka divisions into “Dhaka” in order to make the analysis consistent. The categorization of community literacy level, community level wealth status, and community media exposure into high and low was not available directly in the data set, but generated from maternal education, household wealth index and household media access through a method of aggregation of cluster level [34]. After aggregation of the variables, the categorizations were done based on the median value of the generated variables.

### Statistical analysis

In this study, we analysed data from across the three different surveys and pooled data from the three surveys as well to understand the overall vaccination coverage as well as the changes in vaccination coverage across surveys. We used descriptive statistics to show the characteristics of respondents and the differences in the vaccination coverage between categories were tested using Pearson chi-square analysis. To explore the weighted prevalence of fully vaccination of children across different sub-categories, we used the “svy” command for assigning the sample weight to adjust for clustering effect and sample stratification. Additionally, maps showing the spatial distribution of change rates of full vaccination coverage in the three surveys were created. The change rates of full vaccination coverage over time within each division were calculated using the formula:

$$\frac{{(\left( \begin{array}{l}\% fully\,\\vaccinated\\\,in\,the\,recent\\\,year\end{array} \right) - \left( \begin{array}{l}\% fully\\\,vaccinated\\\,in\,the\,previous\\\,year\end{array} \right))}}{{\left( \begin{array}{l}\% fully\,vaccinated\\\,in\,the\,previous\,year\end{array} \right)}} \times 100$$

Considering the two-stage stratified cluster sampling of BDHSs, we used multilevel (2-level) logistic regression (MLLR) analysis to identify the factors influencing full vaccination coverage by reducing the cluster effects. Since a single-level analysis would not be appropriate for analyzing such data sets that have hierarchical structures [35], we considered the enumeration areas (clusters) as level-2 factor for all the regression models. Both the chi-square and MLLR analysis were executed for each survey year separately as well as for the pooled data. The survey year (as a variable) was considered as confounding factor while conducting the regression analysis on pooled data. Multicollinearity among independent variables was checked using variance inflation factor (VIF). After employing the multilevel models, the intra-class correlation coefficient (ICC) was also estimated to check the cluster effects on the outcome variable. The adjusted odds ratio (AOR) along with 95% confidence interval (CI) were used to interpret the findings and 5% significance level was considered. All analyses were performed using the statistical package SPSS (version 23.0) and STATA (version 17.0). The change rate of full vaccination was also shown in a map which was generated using ArcGIS (version 10.8).

## Results

The current study estimated the vaccination coverage among 12–35 months aged children in Bangladesh over three time periods from the data of BDHS (2011, 2014, and 2017-18). Though in 2014 the coverage of full vaccination (85.13%) is slightly lower than the previous BDHS 2011 (86.17%), the status of full vaccination has increased significantly over time (89.23% in 2017-18) (p < 0.001). The vaccination coverage which was reported either from vaccination cards or by mother recall has been also significantly increased over time. In 2011, the coverage of BCG was 97.33%, in 2014 it was 97.70% and while in 2017-18 the coverage increased to 98.50%. All three doses of polio (Polio 1, Polio2, Polio 3) vaccines were observed to be increased over time and a significant increment (p < 0.001) was seen in the full coverage of OPV (1–3) though a little decrease has been observed in 2014 DHS. Similarly, the full coverage of DTP was also increased from 2011 (92.96%) to 2017-18 (96.01%) (p < 0.001) (Table 1).

**Table 1:** Vaccination coverage estimates (weighted percentage) for children 12–35 months of age by survey year

			2011 (N = 3098)		2014 (N = 3090)		2017-18 (N = 3313)		P value
			n	% (95% CI)	n	% (95% CI)	n	% (95% CI)	
**Vaccination card** ^a^									
Yes, seen			1938	5.06 (4.34,5.89)	2175	69.69 (68.07,71.26)	2355	70.63 (69.06,72.14)	**< 0.001**
Yes, Not seen			811	61.29 (59.56,63.00)	764	25.87 (24.38,27.42)	607	18.71 (17.43,20.07)	
No longer has card			214	26.00 (24.49, 27.58)	51	1.45 (1.09,1.93)	231	7.12 (6.30,8.04)	
No card			134	7.64 (6.76, 8.64)	100	2.98 (2.44,3.63)	120	3.54 (2.97,4.22)	
**Reported vaccinations from vaccination card or mother recall**									
BCG ^a^			3017	97.33 (96.70,97.85)	3000	97.70 (97.12,98.17)	3261	98.50 (98.02,98.86)	**< 0.001**
Polio 1^b^			3024	97.66 (97.07,98.14)	2990	97.48 (96.87,97.97)	3253	98.31 (97.81,98.69)	**< 0.001**
Polio 2^c^			2969	95.82 (95.06,96.48)	2940	96.12 (95.39,96.73)	3210	97.07 (96.44,97.59)	**0.001**
Polio 3^c^			2887	93.25 (92.31,94.08)	2847	92.88 (91.93,93.72)	3135	94.83 (94.03,95.53)	**0.001**
		***Polio vaccination completion (OPV 1–3)*** ^***b***^							
		Full	2886	93.13 (92.18,93.97)	2846	92.78 (91.83,93.63)	3133	94.65 (93.84,95.37)	**< 0.001**
		Partial	138	4.53 (3.85, 5.33)	145	4.74 (4.05,5.53)	121	3.70 (3.11,4.39)	
		None	74	2.34 (1.86, 2.93)	99	2.48 (1.99,3.08)	59	1.65 (1.27,2.14)	
DTP 1^b^			3017	97.45 (96.83,97.95)	2980	96.87 (96.20,97.42)	3257	98.41 (97.93,98.79)	**< 0.001**
DTP 2^d^			2963	95.54 (94.75,96.21)	2963	95.65 (94.89,96.31)	3229	97.54 (96.96,98.01)	**< 0.001**
DTP 3^d^			2883	92.97 (92.01,93.81)	2838	92.40 (91.43,93.27)	3175	96.01 (95.30,96.63)	**< 0.001**
	***DTP vaccination completion (DTP 1–3)*** ^***b***^								
	Full		2882	92.96 (92.00, 93.81)	2838	92.38 (91.41,93.26)	3175	96.01 (95.30,96.63)	**< 0.001**
	Partial		135	4.49 (3.81,5.28)	142	4.48 (3.82, 5.26)	82	2.40 (1.93,2.98)	
	None		80	2.55 (2.05, 3.17)	110	3.13 (2.58,3.80)	56	1.59 (1.21,2.07)	
Measles ^e^			2747	88.66 (87.49, 89.73)	2668	87.22 (86.01,88.33)	3020	91.33 (90.33,92.24)	**< 0.001**
**Vaccination status** ^**f**^									
Full			2694	86.17 (85.46,87.86)	2611	85.13 (83.86,86.33)	2954	89.23 (88.13,90.23)	**< 0.001**
Partial			336	11.13 (10.07,12.29)	399	12.89 (11.78,14.10)	311	9.42 (8.48,10.46)	
None			68	2.16 (1.70,2.74)	80	1.97 (1.54,2.52)	48	1.35 (1.01, 1.80)	

^a^ 2011 n = 3097; 2014 n = 3090; 2017-18 n = 3313

^b^ 2011 n = 3098; 2014 n = 3090; 2017-18 n = 3313

^c^ 2011 n = 3095; 2014 n = 3087; 2017-18 n = 3311

^d^ 2011 n = 3097; 2014 n = 3089; 2017-18 n = 3313

^e^ 2011 n = 3095; 2014 n = 3086; 2017-18 n = 3313

^f^ 2011 n = 3098; 2014 n = 3090; 2017-18 n = 3313

The overall prevalence (weighted) of full vaccination coverage was 86.71% in 2011, 85.13% in 2014, and 89.23% in 2017-18 where the pooled prevalence was 87.06%. The pooled analysis shows that all the variables except sex of children and maternal age were found to be significantly associated with the full vaccination coverage (all p < 0.05). The pooled data also shows that the highest percentage (weighted) of full vaccination was observed among children from Rangpur division (92.28%) followed by Khulna division (90.55%) while the worst situation was found in Sylhet division (Table 2).

**Table 2:** Bivariate distribution of basic vaccination coverage (full) by socio-demographic variables among children aged 12–35 months in Bangladesh

Variables	Fully vaccinated											
	2011 (N = 2694)			2014 (N = 2611)			2017-18 (N = 2954)			Pooled (N = 8259)		
	n	% (95% CI)	p	n	% (95% CI)	p	n	% (95% CI)	p	n	% (95% CI)	p
***Individual and household level variables***												
**Sex of child**												
Female	1339	84.97(83.11,86.65)	**0.005**	1289	86.05(84.22,87.70)	0.426	1414	88.41(86.82,89.83)	0.560	4217	87.08 (86.09,88.01)	0.392
Male	1355	88.51(86.80,90.02)		1322	84.30(82.47,85.97)		1540	90.11(88.55,91.47)		4042	88.05 (86.09,88.01)	
**Current age of child**												
12–23 months	1328	85.94(84.11,87.58)	0.168	1281	83.79(81.92,85.50)	**0.002**	1463	88.27(86.64,89.72)	**0.044**	4072	86.02 (85.01,86.97)	**< 0.001**
24–35 months	1366	87.48(85.73,89.04)		1330	86.54(84.75,88.14)		1491	90.18(88.67,91.52)		4187	88.12 (87.17,89.01)	
**Maternal age**												
< 25years	1436	87.56(85.88,89.07)	0.068	1357	85.34(83.54,86.97)	0.307	1464	88.07(86.45,89.52)	0.102	4257	87.01 (86.05,87.91)	0.061
25–34 years	1079	86.30(84.28,88.10)		1082	84.90(82.88,86.73)		1293	90.47(88.84,91.89)		3454	87.32 (86.25.88.32)	
> 34 years	179	82.08(76.04,86.86)		172	85.04(79.73,89.14)		197	90.21(85.43,93.53)		548	85.87 (82.93,88.38)	
**Maternal education**												
No education	404	75.94(72.24,79.29)	**< 0.001**	295	72.24(67.97,76.15)	**< 0.001**	170	78.60(72.69,83.53)	**< 0.001**	869	75.04 (72.56,77.37)	**< 0.001**
Primary	764	83.41(80.90,85.65)		688	79.89(77.13,82.39)		778	84.71(82.27,86.87)		2230	82.72 (81.27,84.08)	
Secondary	1235	91.69(90.07,93.06)		1303	90.18(88.59,91.58)		1451	91.36(89.89,92.63)		3989	91.05 (90.18,91.85)	
Higher	291	97.05(94.07,98.56)		325	94.50(91.33,96.56)		555	94.73(92.57,96.29)		1171	95.19 (93.77,96.29)	
**Wealth index**												
Poor	1031	82.43(80.31,84.37)	**< 0.001**	964	77.30(74.94,79.49)	**< 0.001**	1210	87.38(85.52,89.03)	**0.001**	3205	82.46 (81.26,83.60)	**< 0.001**
Middle	506	87.92(85.07,90.29)		508	88.70(85.87,91.02)		520	90.14(87.57,92.23)		1534	88.95 (87.42,90.30)	
Rich	1157	91.16(89.36,92.68)		1139	91.39(89.74,92.80)		1224	90.69(89.02,92.14)		3520	91.07(90.12,91.94)	
**Media access**												
Never/less than once	1256	83.70(81.79,85.45)	**< 0.001**	1176	79.25(77.16,81.20)	**< 0.001**	1306	87.43(85.63,89.03)	**0.002**	3738	83.39 (82.28,84.44)	**< 0.001**
At least once a week	1438	89.82(88.19,91.24)		1435	90.69(89.18,92.00)		1648	90.63(89.23,91.86)		4521	90.41 (89.56,91.19)	
**Place of delivery**												
Home	1835	84.15(82.58,85.60)	**< 0.001**	1568	81.73(79.97,83.36)	**< 0.001**	1452	87.02(85.35,88.53)	**< 0.001**	4855	84.16 (83.22,85.07)	**< 0.001**
Health facility	859	93.55(91.67,95.03)		1043	90.85(89.08,92.37)		1502	91.54(90.09,92.80)		3404	91.78 (90.84,92.63)	
**ANC visit**												
No visit	710	79.12(76.39,81.61)	**< 0.001**	484	73.93(70.50,77.09)	**< 0.001**	196	82.12(76.78,86.46)	**< 0.001**	1390	77.61 (75.65,79.45)	**< 0.001**
1 to 3 visits	1064	90.16(88.36,91.71)		1139	86.06(84.13,87.79)		1148	87.33(85.48,88.98)		3351	87.76 (86.71,88.74)	
4 or more visits	737	92.41(90.20,94.16)		842	92.55(90.68,94.07)		1434	92.38(90.93,93.61)		3013	92.44 (91.46,93.31)	
Others^#^	226	81.90(76.43,86.33)		146	82.34(76.49,86.99)		176	87.08(81.70,91.05)		505	83.67 (80.60,86.34)	
**Number of children**												
Single	1018	89.75(87.82,91.41)	**< 0.001**	1065	86.96(84.95,88.74)	**< 0.001**	1147	90.44(88.72,91.92)	0.118	3230	89.07 (88.01,90.04)	**< 0.001**
Two children	867	87.01(84.80,88.94)		860	87.90(85.79,89.73)		1030	89.16(87.24,90.82)		2757	88.08 (86.91,89.15)	
More than two	809	82.93(80.43,85.17)		686	79.53(76.79,82.02)		777	87.52(85.17,89.54)		2272	83.26 (81.82,84.60)	
***Community level variables***												
**Divisions**												
Barisal	304	86.40(80.39,90.78)	**< 0.001**	300	83.81(77.71,88.50)	**< 0.001**	289	87.19(81.42,91.35)	**0.005**	893	85.78 (82.54,88.50)	< 0.001
Chittagong	529	83.12(80.20,85.68)		524	84.45(81.60,86.93)		501	88.46(85.93,90.58)		1554	85.36 (83.80,86.78)	
Dhaka	439	86.39(84.06,88.42)		484	88.18(86.16,89.93)		786	88.50(86.51,90.23)		1709	87.76 (86.58,88.85)	
Khulna	329	91.70(87.91,94.38)		308	87.50(82.85,91.02)		303	92.10(88.38,94.70)		940	90.55 (88.37,92.36)	
Rajshahi	351	90.19(86.87,92.75)		330	86.52(82.29,89.86)		323	91.27(88.06,93.68)		1004	89.54 (87.59,91.20)	
Rangpur	342	91.31(87.64,93.97)		350	93.37(89.96,95.68)		340	92.20(88.92,94.57)		1032	92.28 (90.43,93.80)	
Sylhet	400	81.02(75.47,85.56)		315	63.81(58.15,69.11)		412	85.72(81.05,89.40)		1127	76.30 (73.22,79.12)	
**Place of residence**												
Urban	902	88.78(86.27,90.88)	**0.001**	845	87.77(85.35,89.84)	0.071	1020	88.51(86.27,90.44)	0.920	2767	88.34 (87.01,89.55)	**0.002**
Rural	1792	86.07(84.61,87.41)		1766	84.22(82.70,85.63)		1934	89.49(88.21,90.64)		5492	86.63 (85.82,87.39)	
**Community literacy level of women**												
High	1409	90.82(89.23,92.19)	**< 0.001**	1218	88.72(87.11,90.15)	**< 0.001**	1609	92.01(90.64,93.19)	**< 0.001**	4455	90.89 (9005,91.66)	**< 0.001**
Low	1285	82.98(81.07,84.73)		1393	81.20(79.16,83.09)		1345	86.24(84.47,87.83)		3804	83.14 (82.05,84.18)	
**Community wealth index level**												
High	1376	85.84(84.00,87.49)	**0.017**	1270	80.63(78.64,82.47)	**< 0.001**	1528	88.80(87.24,90.19)	**0.034**	4014	85.16 (84.14,86.12)	**< 0.001**
Low	1318	87.57(85.83,89.13)		1341	89.91(88.30,91.31)		1426	89.70(88.11,91.10)		4245	89.07 (88.14,89.93)	
**Community media exposure**												
High	1455	89.10(87.51,90.51)	**< 0.001**	1174	89.78(88.24,91.14)	**< 0.001**	1508	89.92(88.41,91.25)	**0.020**	4356	89.26 (88.37,90.08)	**< 0.001**
Low	1239	83.92(81.92,85.74)		1437	80.00(77.91,81.94)		1446	88.48(86.83,89.95)		3903	84.62 (83.55,85.64)	
**Total**	2694	86.71(85.46,87.86)		2611	85.13(83.86,86.33)		2954	89.23(88.13,90.23)		8259	87.06 (86.38,87.72)	

^#^ Others included missing values and don’t know,

The bolded p values indicate the statistical significance, CI = Confidence Interval

**Fig. 3:**
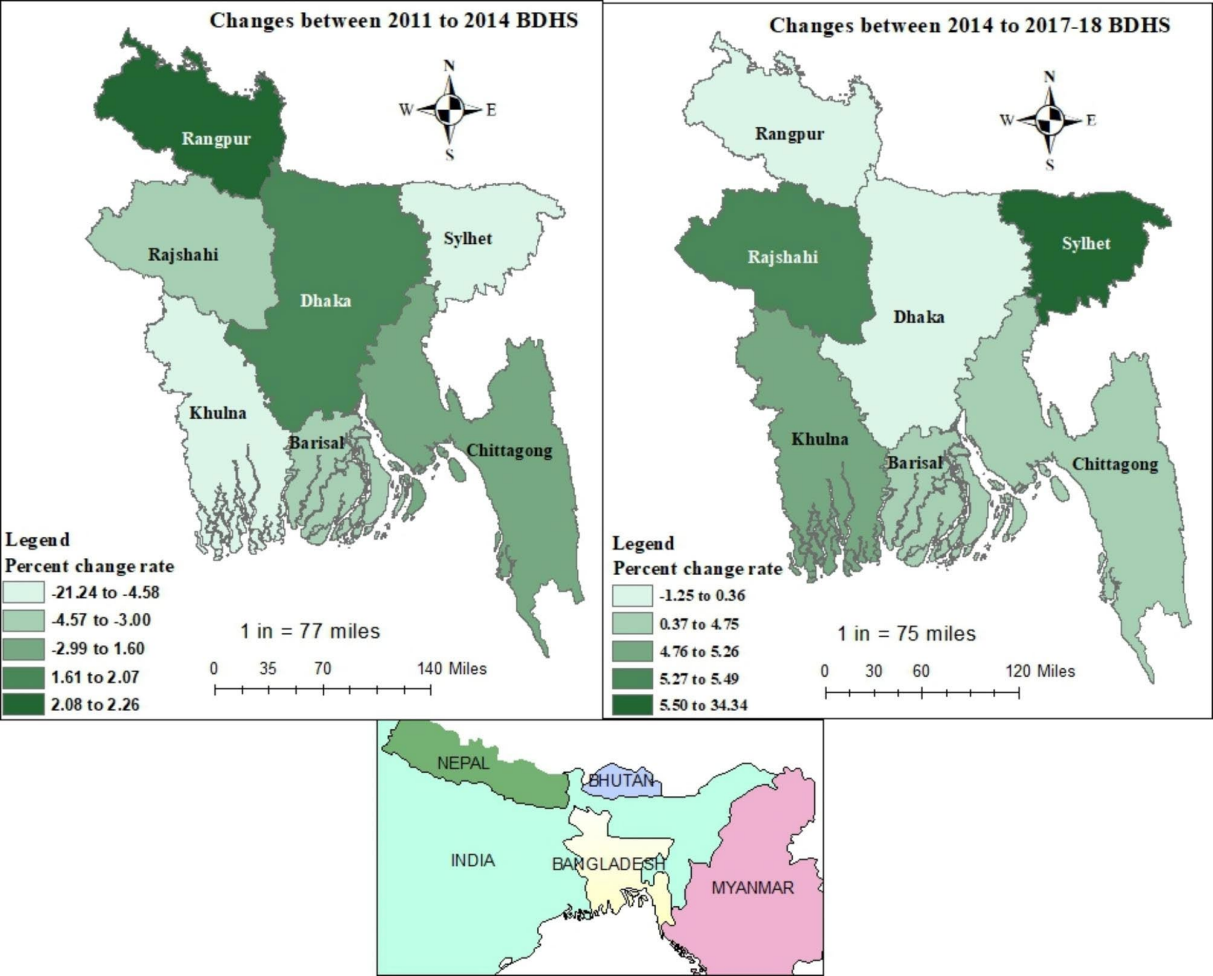
Spatial distribution of change rate in vaccination status among children aged 12–35 months old from BDHS 2011 to 2014 and BDHS 2014 to 2017-18

Figure 3 depicts the geographical pattern of the change rate in full vaccination coverage throughout three survey periods. While most divisions saw a reduction in full vaccination coverage over time from 2011 to 2014, the highest positive change rate from 2011 to 2014 was found in Rangpur division (2.26%) and Sylhet division had the worst scenario (-21.24%). Interestingly, from 2014 to 2017-18, all divisions experienced an increase in vaccination status except Rangpur division (-1.25%), while the highest improvement regarding the change rate was found in Sylhet division (34.34%).

Random-effect parameters of adjusted regression models suggest that clustering variations are present in the outcome measure among enumerations (clusters). Considering the stratified (two-stage) sampling design of the survey, two-level logistic regression analysis approach was employed that allows to remove the clustering effect to ensure precise findings. All variables were included in the adjusted model for controlling the confounding effect of the covariates. The adjusted regression model of pooled analysis demonstrates that age of the child, maternal age and education, ANC visit of the mother, household wealth index, place of residence and community literacy level of women were significantly associated with the coverage of the full vaccination.

We found that children of mothers having secondary, and higher education were more likely to get full vaccination than the mothers who had no formal education in all three waves of BDHS and pooled analysis. Pooled analysis found that children from households with rich wealth index were 27% (AOR = 1.27, 95% CI: 1.04 to 1.55) more likely to get full vaccination compared to those from families with poor wealth index. From the pooled analysis, we also found that if the mother sought at least 4 ANC visits then the likelihood of getting full vaccination was increased by 77% (AOR = 1.77, 95% CI: 1.44 to 2.17) compared to those having no ANC visit. The coverage of full vaccination was higher among the children with mothers aged 25–34 years and above 34 years compared to children of mothers aged < 25 years in 2014 and 2017-18 and in pooled analysis. Children from urban areas were more likely to get fully vaccinated than their rural counterparts and the association is found to be significant in 2014 (AOR = 1.48, 95% CI: 1.06 to 2.06) and in pooled analysis (AOR = 1.25, 95% CI: 1.05 to 1.49). This study also shows that higher community literacy level of women was associated with the higher odds of getting full vaccination in 2011 (AOR = 1.46, 95% CI: 1.04 to 2.06), 2017-18 (AOR = 1.46, 95% CI: 1.03 to 2.05), and in pooled analysis (AOR = 1.36, 95% CI: 1.12 to 1.65). Significant regional variation was also observed in pooled analysis. Children from Rangpur division were more likely to get full vaccination compared to Barisal division (AOR = 1.72. 95% CI: 1.32 to 2.79) (Table 3).

**Table 3:** Multilevel regression analysis showing the factors associated with full vaccination coverage of children aged 12–35 months in Bangladesh

Variables	2011		2014		2017-18		Pooled	
	AOR (95% CI)	p value	AOR (95% CI)	p value	AOR (95% CI)	p value	AOR (95% CI)	p value
***Individual and household level variables***								
**Sex of child**								
Male	Ref		Ref		Ref		Ref	
Female	0.69 (0.55, 0.88)	**0.002**	1.00 (0.80, 1.26)	0.990	1.06 (0.83, 1.35)	0.650	0.93 (0.81, 1.05)	0.241
**Current age of child**								
12–23 months	Ref		Ref		Ref		Ref	
24–35 months	1.31 (1.03, 1.67)	**0.029**	1.63 (1.29, 2.06)	**< 0.001**	1.31 (1.03, 1.69)	**0.031**	1.40 (1.22, 1.59)	**< 0.001**
**Maternal age**								
< 25 years	Ref		Ref		Ref		Ref	
25–34 years	0.91 (0.66, 1.25)	0.553	1.58 (1.15, 2.17)	**0.004**	1.51 (1.09, 2.08)	**0.014**	1.30 (1.09, 1.54)	**0.004**
> 34 years	0.80 (0.48, 1.35)	0.404	2.06 (1.21, 3.51)	**0.008**	1.78 (0.98, 3.22)	**0.059**	1.39 (1.03, 1.87)	**0.031**
**Maternal education**								
No education	Ref		Ref		Ref		Ref	
Primary	1.45 (1.06, 1.99)	**0.020**	1.54 (1.11, 2.13)	**0.010**	1.44 (0.94, 2.22)	0.094	1.53 (1.27, 1.84)	**< 0.001**
Secondary	2.31 (1.59, 3.34)	**< 0.001**	2.11 (1.47, 3.03)	**< 0.001**	2.36 (1.49, 3.74)	**< 0.001**	2.39 (1.94, 2.94)	**< 0.001**
Higher	7.28 (3.05, 17.39)	**< 0.001**	4.29 (2.22, 8.32)	**< 0.001**	3.79 (2.05, 7.02)	**< 0.001**	4.51 (3.15, 6.45)	**< 0.001**
**Wealth index**								
Poor	Ref		Ref		Ref		Ref	
Middle	1.14 (0.81, 1.61)	0.437	1.10 (0.79, 1.54)	0.574	1.09 (0.74, 1.59)	0.667	1.11 (0.92, 1.35)	0.266
Rich	1.46 (1.01, 2.12)	**0.045**	1.45 (1.01, 2.12)	**0.049**	1.00 (0.68, 1.47)	0.998	1.27 (1.04, 1.55)	**0.020**
**Media access**								
Never/ less than once	Ref		Ref		Ref		Ref	
At least once a week	0.86 (0.64, 1.15)	0.309	1.35 (0.99, 1.82)	0.053	0.90 (0.67, 1.22)	0.508	1.07 (0.91, 1.26)	0.412
**Place of delivery**								
Home	Ref		Ref		Ref		Ref	
Health facility	1.44 (1.01, 2.04)	**0.042**	0.90 (0.68, 1.20)	0.480	1.28 (0.96, 1.70)	0.087	1.15 (0.97, 1.35)	0.103
**ANC visit**								
No visit	Ref		Ref		Ref		Ref	
1 to 3 visits	1.42 (0.96, 2.10)	0.079	1.49 (1.12, 1.99)	**0.007**	1.13 (0.74, 1.73)	0.580	1.41 (1.19. 1.66)	**< 0.001**
4 or more visits	1.52 (1.14, 2.03)	**0.005**	1.73 (1.19, 2.50)	**0.004**	1.63 (1.01, 2.61)	**0.044**	1.77 (1.44, 2.17)	**< 0.001**
Others^#^	0.90 (0.57, 1.40)	0.635	1.26 (0.77, 2.07)	0.348	1.22 (0.66, 2.26)	0.521	1.09 (0.83, 1.43)	0.533
**Number of children**								
Single	Ref		Ref		Ref		Ref	
Two	0.89 (0.64, 1.23)	0.465	1.02 (0.75, 1.39)	0.900	0.94 (0.68, 1.30)	0.702	0.97 (0.81, 1.16)	0.730
More than two	1.13 (0.76, 1.69)	0.553	0.63 (0.43, 0.93)	**0.021**	0.89 (0.58, 1.38)	0.612	0.86 (0.69, 1.08)	0.195
***Community level variables***								
**Divisions**								
Barisal	Ref		Ref		Ref		Ref	
Chittagong	0.79 (0.47, 1.32)	0.364	1.01 (0.60, 1.68)	0.967	0.99 (0.56, 1.75)	0.983	0.91 (0.66, 1.25)	0.555
Dhaka	1.03 (0.60, 1.79)	0.902	1.05 (0.62, 1.79)	0.845	1.17 (0.69, 1.98)	0.562	1.21 (0.90, 1.62)	0.209
Khulna	1.35 (0.72, 2.54)	0.353	0.74 (0.42, 1.30)	0.292	1.32 (0.67, 2.59)	0.424	1.04 (0.73, 1.49)	0.819
Rajshahi	1.72 (0.94, 3.15)	0.078	1.33 (0.76, 2.35)	0.317	1.43 (0.75, 2.70)	0.276	1.62 (1.13, 2.31)	**0.008**
Rangpur	1.51 (0.83, 2.73)	0.177	2.10 (1.16, 3.81)	**0.015**	1.65 (0.86, 3.15)	**0.133**	1.72 (1.32, 2.79)	**0.041**
Sylhet	0.85 (0.50, 1.46)	0.556	0.37 (0.23, 0.61)	**< 0.001**	0.98 (0.55, 1.73)	0.938	0.97 (0.65, 1.45)	0.883
**Place of residence**								
Urban	1.10 (0.77, 1.56)	0.596	1.48 (1.06, 2.06)	**0.021**	1.24 (0.88, 1.74)	0.217	1.25 (1.05, 1.49)	**0.012**
Rural	Ref		Ref		Ref		Ref	
**Community literacy level of women**								
High	1.46 (1.04, 2.06)	**0.030**	1.19 (0.86, 1.64)	0.293	1.46 (1.03, 2.05)	**0.032**	1.36 (1.12, 1.65)	**0.002**
Low	Ref		Ref		Ref		Ref	
**Community wealth index level**								
High	1.21 (0.85, 1.73)	0.294	0.85 (0.60, 1.22)	0.390	0.96 (0.65, 1.42)	0.845	1.03 (0.84, 1.27)	0.747
Low	Ref		Ref		Ref		Ref	
**Community media exposure**								
High	1.19 (0.83, 1.70)	0.353	1.00 (0.70, 1.42)	0.992	0.96 (0.66, 1.40)	0.826	1.02 (0.83, 1.26)	0.842
Low	Ref		Ref		Ref		Ref	
***Random-effect parameters***								
Cluster effects (95% CI)	0.78 (0.60, 1.01)	**< 0.001**	0.79 (0.62, 0.99)	**< 0.001**	0.88 (0.69, 1.12)	**< 0.001**	0.59 (0.50, 0.70)	**< 0.001**
ICC (95% CI)	0.16 (0.09, 0.23)		0.16 (0.11, 0.23)		0.19 (0.13, 0.28)		0.10 (0.07, 0.13)	
AIC	2198.66		2364.61		2165.996		6794.70	

^#^ Others included missing values and don’t know,

The bolded p values indicate the statistical significance, AOR = Adjusted Odds ratio, CI = Confidence Interval

ICC = Intraclass Correlation Coefficient; AIC = Akaike’s Information Criterion

^a^ Significance of cluster random effects is assessed using log-likelihood ratio test (LR test vs. logistic model)

## Discussion

The Sustainable Development Goal encourages countries and governments to take steps to ensure that their national immunization programs are fully vaccinated by 2030 [36]. As a result, understanding the factors that determine vaccination coverage over time is crucial for Bangladesh to assess its progress toward universal childhood immunization. The goal was to track the vaccination status of children aged 12 to 35 months and look at the factors that influence full immunization coverage.

Overall, our findings show that the percentage of those who have had complete vaccination has risen significantly over time. This is substantiated by a recent Bangladeshi study that demonstrated a slight increase in complete immunization coverage over time [16]. However, this increasing change was not linear as we observed a decline in vaccine coverage between 2011 and 2014. The political turmoil that Bangladesh experienced in 2013–2014 may be the cause of a sudden decline in vaccine coverage between the periods. A study conducted in 2014 [37] reported that Bangladesh was dealing with political unrest and volatility at that time, which frequently escalate into violence. Such political unrest, which is frequently accompanied by street violence and the damage of both public and private property, has a serious negative effect on the economy. Such political unrest indirectly affects the health system [37]. The apparent increase in vaccination coverage could, therefore, be attributed to random factors in a certain year rather than a direct result of a long-term consistent increase. Despite the fact that full vaccination status for all vaccines increased significantly over time, BCG continuously had the greatest full vaccination coverage. This is consistent with the findings of Boulton et al., who found that BCG had the highest full vaccination coverage compared to the other vaccinations [16]. This observation is explained by the fact that, unlike other vaccines that are given after a few weeks (such as OPV and DTP) or months (such as measles), BCG is given at birth, minimizing the risk of not getting immunized [38].

We also observed some divisional variations from our spatial analysis of the distribution of change rate in relation to childhood vaccination coverage. A positive change rate was observed in Rangpur division while the worst situation was found in Sylhet division. This is in agreement with the findings of Sheikh et al. [14]. This higher likelihood of incomplete vaccination in the Sylhet division could be linked to the remote hilly and riverine nature of the area coupled with the fragile communication system of this area [14]. Between 2014 to 2017-18, all divisions experienced an increase in vaccination status except Rangpur division. However, the absolute vaccination coverage was highest in the Rangpur division. This calls for further research to understand the divisional variations with respect to vaccination coverage in Bangladesh.

The study found a positive significant association between the current age of the child and full vaccination coverage. Thus, older children were more likely to be fully vaccinated. The findings align with evidence from DR Congo [1]. This is expected because childhood vaccinations are scheduled and for that matter, older children will be at a higher likelihood of being fully vaccinated. Also, Bangladesh has had a fair share of mass vaccination programs over the years [39]. Such mass vaccinations, in the perspective of Alfonso et al. [1], lead to catch-up vaccination with age, thereby predisposing older children (24–35 months) to a higher possibility of being fully vaccinated compared to those aged 12–23 months.

As expected, our findings showed that maternal age and education had a significantly positive association with vaccination coverage over time. This result is substantiated by earlier studies conducted in Bangladesh [40, 41], DR Congo [42], and Ethiopia [4]. These findings indicate that young women and those without formal education are high risk populations where incomplete vaccination is likely to abound. Therefore, it is imperative for policies and interventions that aim to improve childhood vaccination uptake to target this at-risk population. For younger women, the implementation on adolescent and youth friendly health services would be significant in improving vaccination uptake and coverage.

At the contextual level, urban residence, and attending at least 4 ANC visits were significantly associated with increased full vaccination coverage. Similar findings have been reported in Bangladesh [1], as well as studies from Ethiopia [43] and Ghana [44]. Attending at least 4 ANC visits provide mothers with the opportunity to be exposed to more health education regarding the importance of ensuring full vaccination of their children, as well as gain satisfaction with healthcare access which could potentially translate into higher vaccination coverage [45]. Concerning the rural-urban differences in our findings, it highlights a need for the Bangladeshi government to bridge the rural-urban disparities by setting up more community health centers in rural areas.

As expected, the present study showed that children born to rich wealth indexed households were more likely to receive full vaccination as compared to those born into poor wealth indexed households. This is consistent with previous studies [14, 46], and underscores the importance of pro-poor health interventions in bridging the wealth status disparities in childhood vaccination coverage. The study also revealed that higher community literacy level of women was associated with the higher odds of getting full vaccination. This observation can be attributed to the fact that vaccination is influenced by community and societal norms [47].

## Strengths and limitations

Our study has several strengths. The dataset used in this study was nationwide in nature, allowing the findings to be extended to children all over Bangladesh. Furthermore, the DHS dataset is a thoroughly tested, repeatable, standardized, and comprehensive survey. Examining variations in vaccination coverage across surveys and comparing them to an analysis of the aggregated data from all the surveys provides a holistic picture of the country’s vaccine coverage. This, together with the application of stringent statistical processes, ensures the validity of our findings and the study’s replicability in other settings. Nonetheless, there are some inherent limitations to the study that should be considered when interpreting our findings. Because the BDHS uses a cross-sectional design, causality cannot be proven. Furthermore, information on the status of child vaccination is based on either immunization cards or women’s self-reports; hence, recall bias may exist, resulting in an under- or overestimation of vaccination coverage. Also, important residual confounders such as cultural norms and beliefs, and complexities in the supply chain of vaccines could not be assessed in the study as the data set does not have variables to measure their effect on childhood vaccination coverage. Data were taken from the BDHS for 2017–2018, and afterward, the Covid-19 pandemic occurred, which had a major impact on the country’s immunization coverage. The results of this research therefore only represent childhood immunization trends prior to the COVID-19 pandemic, and we were unable to account for the COVID-19’s effects on Bangladesh’s childhood immunization coverage in this study.

## Conclusion

While immunization coverage has risen over time (from 2011 to 2017/18), the increase was not linear as a decrease was seen in some areas from 2011 to 2014. The apparent increase in vaccination coverage could therefore be attributed to random factors in a certain year rather than a direct result of a long-term consistent increase. Based on the significant findings of this study, childhood vaccine interventions should focus on mothers with no formal education, younger mothers (less than 24 years), and children born to mothers in impoverished families to close the gap in full childhood immunization in Bangladesh. In order to attain full childhood immunization, the government must also promote ANC attendance and community literacy.

## Data Availability

The datasets of BDHS 2011, 2014 and 2017-18 are available upon request in the following website: http://dhsprogram.com/data/available-datasets.cfm.
